# Pedunculated small bowel lipoma with heterotopic pancreas causing intussusception

**DOI:** 10.1002/ccr3.1710

**Published:** 2018-07-10

**Authors:** Marianna Papageorge, Abhijeet Waghray, Lilian Chen, James Yoo

**Affiliations:** ^1^ Tufts University School of Medicine Boston Massachusetts; ^2^ Division of Gastroenterology Tufts Medical Center Boston Massachusetts; ^3^ Department of Surgery Tufts Medical Center Boston Massachusetts

**Keywords:** abdominal pain, heterotopic pancreas, intussusception, small bowel lipoma

## Abstract

Small bowel intussusception is a relatively uncommon cause of abdominal pain. The diagnosis is often delayed due to vague symptoms and limitations with current endoscopic and radiographic approaches to evaluate the small bowel lumen. Treatment often requires surgical resection, which can usually be performed in a minimally invasive fashion.

A 36‐year‐old man presented with 4 months of intermittent and progressive abdominal pain and bloating. Laboratory data including stool studies were negative. A CT scan of the abdomen/pelvis (Figure 1A) demonstrated an 8‐cm tubular structure in the terminal ileum with a central fat density and peripheral mucosal covering. However, an abdominal ultrasound, esophagogastroduodenoscopy, nuclear medicine technetium‐99 m scan, and colonoscopy were all normal. A capsule endoscopy study did not visualize the mass. He was referred to a tertiary medical center for further evaluation.

**Figure 1:**
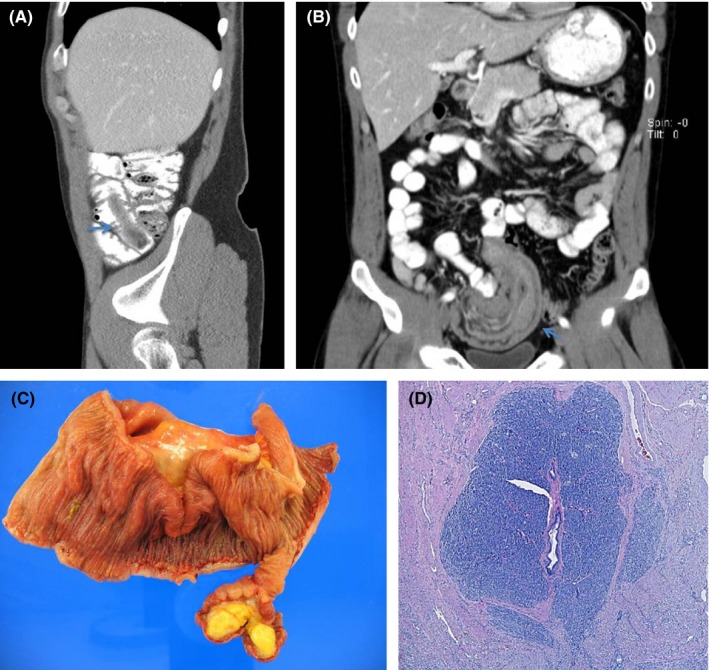
Radiographic images (Figure 1A and Figure 1B), gross pathology (Figure 1C), and histologic evaluation (Figure 1D) demonstrating heterotopic pancreatic tissue within the small bowel lipoma.

Given his persistent symptoms, a CT scan of the abdomen/pelvis was repeated and now revealed a pedunculated ileal mass with intussusception and bowel wall thickening with small bowel obstruction and proximal bowel dilatation (Figure 1B). The differential diagnosis included a small bowel adenoma, adenocarcinoma, Meckel's diverticulum, GIST, lipoma, carcinoid tumor, leiomyoma, and lymphoma. The patient underwent a laparoscopic small bowel resection, performed in an oncologic fashion with wide margins and a lymphadenectomy, because malignancy could not be excluded based on the preoperative evaluation. The patient recovered well with complete resolution of symptoms on follow‐up.

Pathologic examination demonstrated an ileoileal intussusception secondary to a 6.5 cm submucosal lipoma (Figure 1C) with a 3 mm focus of heterotopic pancreas (Figure 1D) with ducts and foci of islet cells. The incidence of pancreatic heterotopias ranges from <1% to 13% with the majority of cases reported in the stomach. Involvement of the small bowel, colon, gallbladder, and mesentery has rarely been reported.^1^, ^2^ In general, patients with clinically asymptomatic pancreatic heterotopia can be observed, with definitive surgical management reserved for symptomatic patients or those that develop complications.

## CONFLICT OF INTEREST

None declared.

## AUTHORSHIP

MP: participated in manuscript writing, literature review, and collection of images. AW: participated in collection of images and manuscript review. LC: participated in manuscript review. JY: participated in manuscript writing and review. All authors contributed equally to acquisition of data, drafting, and revision of the manuscript.
